# Acetylation of Response Regulator Protein MtrA in *M. tuberculosis* Regulates Its Repressor Activity

**DOI:** 10.3389/fmicb.2020.516315

**Published:** 2021-01-15

**Authors:** Krishna Kumar Singh, P. J. Athira, Neerupma Bhardwaj, Devendra Pratap Singh, Uchenna Watson, Deepak Kumar Saini

**Affiliations:** ^1^Department of Molecular Reproduction, Development and Genetics, Indian Institute of Science, Bengaluru, India; ^2^Molecular Biophysics Unit, Indian Institute of Science, Bengaluru, India; ^3^Department of Studies in Zoology, University of Mysore, Mysore, India; ^4^Centre for Biosystems Science and Engineering, Indian Institute of Science, Bengaluru, India

**Keywords:** acetylation, phosphorylation, DNA binding protein, mycobacteria, response regulator, acetyl phosphate

## Abstract

MtrA is an essential response regulator (RR) protein in *M. tuberculosis*, and its activity is modulated after phosphorylation from its sensor kinase MtrB. Interestingly, many regulatory effects of MtrA have been reported to be independent of its phosphorylation, thereby suggesting alternate mechanisms of regulation of the MtrAB two-component system in *M. tuberculosis*. Here, we show that RR MtrA undergoes non-enzymatic acetylation through acetyl phosphate, modulating its activities independent of its phosphorylation status. Acetylated MtrA shows increased phosphorylation and enhanced interaction with SK MtrB assessed by phosphotransfer assays and FRET analysis. We also observed that acetylated MtrA loses its DNA-binding ability on gene targets that are otherwise enhanced by phosphorylation. More interestingly, acetylation is the dominant post-translational modification, overriding the effect of phosphorylation. Evaluation of the impact of MtrA and its lysine mutant overexpression on the growth of H37Ra bacteria under different conditions along with the infection studies on alveolar epithelial cells further strengthens the importance of acetylated MtrA protein in regulating the growth of *M. tuberculosis*. Overall, we show that both acetylation and phosphorylation regulate the activities of RR MtrA on different target genomic regions. We propose here that, although phosphorylation-dependent binding of MtrA drives its repressor activity on *oriC* and *rpf*, acetylation of MtrA turns this off and facilitates division in mycobacteria. Our findings, thus, reveal a more complex regulatory role of RR proteins in which multiple post-translational modifications regulate the activities at the levels of interaction with SK and the target gene expression.

## Introduction

One of the primary signaling systems found in bacteria is two-component signaling (TCS), which contains distinct sensory and regulatory components or proteins. The communication relay between these proteins occurs via a unique phosphorylation design wherein external stimulus causes the autophosphorylation of the sensor kinase (SK) protein, followed by phosphorylation of the response regulator (RR) protein by a phosphotransfer reaction. In this reaction, the phosphoryl group from the SK is transferred to the RR without involving fresh ATP hydrolysis. The phosphorylation of RR has been shown to alter its downstream activity, which generally involves changes in its DNA binding activity (Bourret and Silversmith, 2010; Bretl et al., 2011). This stimulus-to-response cascade, thus, essentially relies on the changes in the phosphorylation status of the RR protein. Over the years, with the advent of more sensitive techniques for identifying post-translation modifications on proteins, other moieties, such as acetylation, have also been reported on the RR proteins (Christensen et al., 2019b; Macek et al., 2019). Similar to phosphorylation (Gao et al., 2007), acetylation has also been shown to affect transcriptional activities of RR proteins (Kim and Yang, 2011; Thao et al., 2011; van Noort et al., 2012; Hu et al., 2013), thereby linking signaling changes to metabolic perturbations in the bacterium (Singh et al., 2019).

Acetylation profiling studies reveal widespread acetylation of signaling proteins in *M. tuberculosis*, which includes TCS proteins and mainly RRs (Liu et al., 2014; Xie et al., 2015; Ren et al., 2016, 2017; Bi et al., 2018; Yang et al., 2018). Of 16 RRs present in the *M. tuberculosis* genome, mass spectrometric analysis has revealed acetylation in 8 RRs. However, no significant impact of acetylation on the activity of RRs has been reported except in two articles published recently. In the first, it is shown that the acetylation of RRs (TcrX and MtrA) affects their interaction with SKs, thus, tuning the fidelity of the SK-to-RR phosphotransfer reaction (Singh et al., 2019). In the other report, deacetylation of the DosR response regulator is shown to promote the hypoxia response in *Mtb*, resulting in much lower infection in mice (Bi et al., 2018; Yang et al., 2018). Based on the scheme of reactions in the TCS signaling process, the effect of acetylation of RR could be recorded at three distinct steps, (i) interaction of SK and RR proteins, (ii) phosphotransfer reaction from SK to RR, and (iii) DNA (or RNA) binding activity of the RR protein.

Essential RR MtrA (Zahrt and Deretic, 2000) is shown to be acetylated in many recent studies (Xie et al., 2015; DeJesus et al., 2017; Singh et al., 2019), and acetylation promotes its interaction with its promiscuous and non-essential cognate SK MtrB (Singh et al., 2019). In the present study, we probe the role of acetylation in regulating various activities of the RR MtrA and identify which post-translational modification from acetylation and phosphorylation is dominant. We establish that acetylation could occur non-enzymatically through acetyl phosphate (AcP). The AcP pool is associated with growth adaptation during energy deprivation (Wolfe, 2005), and it can serve as both a phosphoryl and an acetyl group donor (Schilling et al., 2015). This becomes even more interesting as MtrA controls Mycobacterial replication, reactivation, and cell division by regulating the expression of genes, such as resuscitation promoting factor (*rpfB*) (Sharma et al., 2015) and origin of replication (*oriC*) (Purushotham et al., 2015). In *Mtb* bacterial chromosome replication results in the proliferation of bacilli inside the host during disease progression. This process initiates on a specific site on the bacterial chromosome, also known as the *oriC*, where DnaA, the initiator protein, binds and activates replication followed by cell division (Qin et al., 1999). RpfA–E are hydrolytic enzymes, which are necessary for bacilli resurrection from dormancy during chronic infection of *Mtb* (Sharma et al., 2015) and are crucial for cell wall growth (Davies et al., 2008). This evidence proposes that MtrA acetylation may link the regulatory role of acetyl phosphate in cell division and reactivation of *M. tuberculosis*. Along this line, we verified the effect of acetylated response regulator MtrA (by AcP) for its promoter binding ability on the *oriC* and *rpfB* promoters. In view of the extensive regulatory role of phosphorylation and acetylation in different physiological processes inside the cell, it is tempting to speculate that the combinatorial effect of both PTMs may control the functionality of the MtrA response regulator, which is what we observed in our studies.

Overall, our findings reveal the existence of multimodal regulation of TCS networks wherein multiple cues, internally arising from metabolism, such as acetylation, and externally from the sensor kinase, drive expression of genes that affect cellular physiology, growth, and metabolism. More importantly, we show that nutritional cues dominate the regulatory process governed by RR MtrA, specifically in fine-tuning the bacterial proliferation and division *in vitro* and *in vivo*.

## Materials and Methods

Chemicals, media, biochemicals, and protein reagents were procured from Merck (St. Louis, United States); the antibiotics and DTT (Dithiothreitol) were from Goldbio (United States); protein markers were from Abcam (United Kingdom); and Ni^2+^-NTA resin from Merck (St. Louis, United States). Restriction enzymes were from Thermo Fisher (United States). Cloning primers were from Bioserve (India), and radioactive γ^32^P ATP (>4,000 Ci/mmol) was obtained from BRIT-Jonaki (Hyderabad, India).

### Bacterial Strains and Recombinant Plasmid Construction

Cloning and protein overexpression were carried out in *E. coli* DH10β and BL21 Arctic Express^TM^ (Agilent Technologies, United States) (Ferrer et al., 2003), respectively. The strains were grown in LB medium with 100 μg/ml ampicillin or 50 μg/ml gentamycin as required. The primers used for PCR and cloning are listed in the Supplementary Table. Recombinant plasmids used for protein overexpression have been reported previously (Nambi et al., 2010; Agrawal et al., 2015). Recombinant plasmids for expressing chimeric MtrB-GFP protein, the nucleotide region from the 851 to 1754 bp region of *mtrB*, capable of coding for the 300 aa long cytosolic catalytic domain of the MtrB protein, was PCR amplified from H37Rv genomic DNA using specific primers and cloned in pProEx-Htc vector at *Nco*I and *Bam*HI restriction sites. The *gfp* gene was cloned between *Bam*HI and *Xho*I sites. Similarly, for MtrA-RFP chimeric protein, the *mtrA* gene was cloned in *Nco*I and *Bam*HI restriction sites, and the *rfp* gene was cloned between *Bam*HI and *Xho*I sites in the pPRoEx-Htb expression vector. For in cellulo and *in vivo* analysis, the gene coding for wild-type MtrA protein was cloned in pMV261 vector between *Pst*I and *Hin*dIII sites, and the recombinant plasmid was introduced in *M. tuberculosis* H37Ra for further analysis. Various lysine mutations were introduced by a quick-change, site-directed mutagenesis technique directly on pPROEx or pMV261 plasmids. All the recombinant constructs were verified by DNA sequencing.

### Protein Expression and Purification

The recombinant proteins were expressed and purified as per protocols reported previously (Nambi et al., 2010; Agrawal et al., 2015, 2016).

### Phosphorylation Assays

Autophosphorylation and phosphotransfer assays were performed as per protocols described previously (Agrawal et al., 2015; Singh et al., 2019). Brief protocols are described in the Supplementary Material.

### Acetylation Status Analysis and Site Identification by MS/MS Analysis

Acetylation analysis of the test proteins was performed by Western blotting using anti-acetyl lysine antibodies as previously described (Singh et al., 2019).

### Determination of AcP Binding Affinity to RR by Microscale Thermophoresis (MST) Analysis

MST measurement to determine the affinity between the SK-RR proteins has been described previously (Singh et al., 2019), and the same was adapted for this study (RR-AcP).

### Electrophoretic Mobility Shift Assay (EMSA)

A 526-bp region of the *oriC* and 440-bp of *rpfB* promoter region were PCR-amplified from an *M. tuberculosis* H37Rv genomic DNA template using specific primers as reported previously (Purushotham et al., 2015; Sharma et al., 2015) (Supplementary Table). The PCR products were purified and end-labeled with ^32^P using T4 polynucleotide kinase (Thermo Scientific, USA) as per the manufacturer’s protocol. After labeling, the fragments were purified and used for the EMSA described previously (Singh et al., 2019).

### Fluorescence Resonance Energy Transfer (FRET)

FRET analysis between SK and RR proteins was performed as previously reported (Agrawal et al., 2016), and the protocol is described in brief in the Supplementary Material.

### Phosphorylation Analysis of MtrA With Acetyl Phosphate

Radiolabeled acetyl phosphate was generated using the protocol described previously (Molle and Buttner, 2000). In brief, ^32^P-labeled acetyl phosphate was generated by an enzymatic reaction using acetate kinase (Sigma) and 10 μCi [γ^32^P] ATP in a reaction buffer containing 25 mM Tris-HCl (pH 7.5), 60 mM potassium acetate, and 10 mM MgCl_2_ for 15 min at 25°C. The reaction mixture was then filtered through a 3 kDa membrane filter to remove the acetate kinase from the reaction mix to obtain labeled AcP. The flow-through containing ^32^P-AcP was incubated with 150 pmoles of MtrA or DevR as a positive control (Agrawal et al., 2015) for 15 min at 30°C. The reaction mixture was terminated by adding a 1x SDS buffer and analyzed by SDS-PAGE, followed by autoradiography.

### Circular Dichroism Spectroscopy

MtrA protein with or without AcP was subject to circular dichroism spectroscopy using a JASCO J-810 Spectropolarimeter (Singh et al., 2019). Spectra for 100 μg of the MtrA protein (in 1 × PBS) was analyzed between wavelength 190–300 nm to record protein secondary structures.

### Generation of *E. coli* Δ*pta* and Δ*ackA* Knockout Strains

The knockout strains of *E.coli* K12 for *pta* and *ackA* genes were obtained from C. Choudhary (University of Copenhagen) (Weinert et al., 2013). They were used as donors in the P1 transduction experiment. The cultures of these strains were infected with wild-type P1 lytic phage and plated at 37°C for plaque formation. Plaques were recovered using 1M MgCl_2_-CaCl_2_ (MC) buffer at 4°C for 3–4 h. Donor phage was transduced in BL21 Arctic cells, and the transduced cells were then washed with 1M sodium citrate buffer to terminate transduction. The transduced cells were revived in LB for 30 min at 37°C and plated on LA containing kanamycin or gentamycin depending on the donor phenotype.

### *In vivo* Acetylation Analysis in *E. coli*

To determine if MtrA is acetylated by AcP *in vivo*, the phosphotransacetylase (Δ*pta*) and acetate kinase (Δ*ackA*) mutant *E. coli* BL21 Arctic strain were transformed with an MtrA expression plasmid. MtrA protein expression and purification were performed as per the protocol described above. The acetylation status of the MtrA protein was analyzed by Western blotting as per the protocol described in Supplementary Material.

### Growth Analysis and Macrophage Infection Studies

H37Ra strains carrying the wild type of lysine mutants (described above) of MtrA in pMV261 were grown in Middlebrook 7H9, Tween-80, OADC media. The primary culture was grown to mid-log phase and sub-cultured in either rich (7H9 + OADC) or poor (7H9 alone without OADC) media with 25 μg/ml kanamycin. The OD_600_ of the culture was recorded at various time points (as shown). For infection studies, the A549 alveolar epithelial cell line was infected with various strains, and colony forming units (cfu) at 4 and 24 h post-infection (hpi) were examined.

### Gene Expression Analysis

Analysis of changes in the expression levels of the *rpfB* gene was done from various strains by quantitative RT-PCR analysis as per the protocol previously described (Singh et al., 2019). The strains were grown in poor media and harvested at day 15 for expression analysis.

Additional details for all the protocols are described in the Supplementary Material.

## Results

To probe the impact of acetylation on RR proteins, we chose to examine the effect on an essential RR MtrA of *M. tuberculosis*. This specific RR regulates critical physiological processes in mycobacterial cells, including cell division, cell length control, etc. (Fol et al., 2006; Rajagopalan et al., 2010; Purushotham et al., 2015). Although the RR MtrA is essential, its cognate sensor kinase MtrB is not essential, and it participates in significant crosstalk (Fol et al., 2006; Agrawal et al., 2015; Banerjee et al., 2019; Singh et al., 2019). Given this role, we aimed to understand the impact of acetylation on the activities of the RR MtrA and understand the synchrony, if any, in the phosphorylation mediated activity and that of acetylation-mediated regulation.

### RR MtrA Is Acetylated Enzymatically as Well as Non-enzymatically by AcP

**FIGURE 1:**
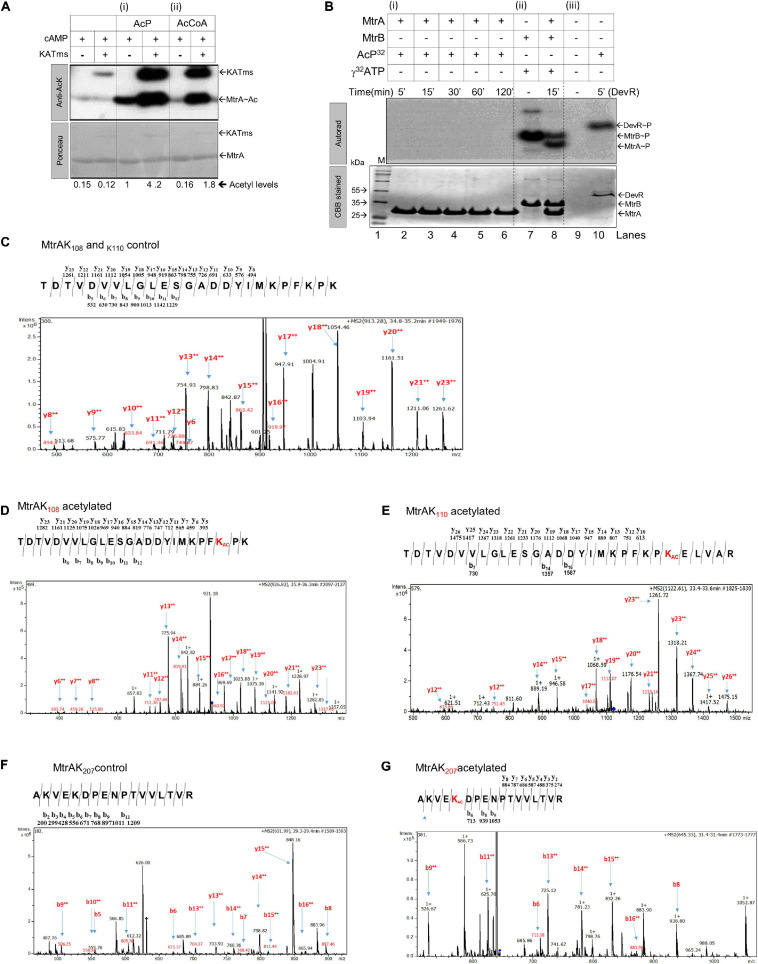
Analysis of acetylation or phosphorylation by AcP on MtrA protein. **(A)** Acetylation of purified MtrA protein using AcP or acetyltransferase (KATms, MSMEG_5458) enzyme from *M. smegmatis* (Singh et al., 2019). Acetylation was detected using an anti-acetyl antibody by Western blotting, top (KATms, acetyltransferase from *M. smegmatis*). Bottom, Ponceau stained blot (M, marker). Numbers indicate a quantitative measurement of acetylation on the respective MtrA protein. The amount of signal recorded for acetylated MtrA was taken as 1, and the rest were normalized to that (*n* = 3). **(B)** Analysis of AcP as a phosphodonor for MtrA. ^32^P labeled AcP was synthesized, and an *in vitro* autophosphorylation reaction was performed as described in materials and methods. Reactions were analyzed on 15% SDS-PAGE, followed by autoradiography. Lane 1, Marker; lane 2–6, MtrA protein with labeled AcP for indicated time points; lane 7, autophosphorylated MtrB; lane 8, MtrB with MtrA incubated with ATP for 15 min, and lane 10, RR DevR used as a positive control. The top panel, autoradiogram, and lower panel correspond to CBB stained gel (*n* = 3). **(C)** MS/MS analysis spectra for MtrA K_108_ and MtrA K_110_ peptide. **(D)** MS/MS analysis spectra for acetylated MtrA K_108_. **(E)** MS/MS analysis spectra for acetylated MtrA K_110_. **(F)** MS/MS analysis spectra for MtrA K_207_ peptide. **(G)** MS/MS analysis spectra for acetylated MtrA K_207_.

Previously, enzymatic acetylation of RR MtrA has been reported (Singh et al., 2019); we reiterated the same using acetyl CoA as a donor molecule of the acetyl group and acetyltransferase (KATms) as the enzyme catalyzing the reaction (Singh et al., 2019). As anticipated, robust acetylation of MtrA was detected (Figure 1A, right panel) (Singh et al., 2019). We also probed for non-enzymatic acetylation directly through AcP as observed earlier for the PhoP RR of *Salmonella* typhimurium (Ren et al., 2019). In this experiment, distinct acetylation of MtrA was recorded (Figure 1A, middle panel), comparable to the enzymatically catalyzed reaction (Figure 1A, last lane). When we incubated MtrA with acetyltransferase and AcP, we recorded even higher acetylation for MtrA (Figure 1A, lane 4), revealing that RR MtrA acetylation could be a mix of non-enzymatic as well as enzymatic acetylation (through KATms). We also observed acetylation of the KATms enzyme through AcP similar to that from Acetyl-CoA (Nambi et al., 2010). Our finding shows that acetylation of RR could occur directly from AcP independent of the catalyzing enzyme/s (Figure 1A, lane 3).

Given that AcP is used as a phosphodonor species for RR proteins (Lukat et al., 1992; McCleary and Stock, 1994; Wolfe, 2005, 2010; Klein et al., 2007; Kuhn et al., 2014), we also tested if it can serve as both a phosphodonor and an acetyl group donor. Toward this, ^32^P-labeled AcP, synthesized enzymatically using acetate kinase (Molle and Buttner, 2000), was used to probe phosphorylation of MtrA if any. As a control, another RR of *Mtb*, DevR, known to be phosphorylated by AcP (Agrawal et al., 2015; Sharma et al., 2019), was used. Although we recorded distinct phosphorylation for the DevR protein (Figure 1B, panel iii), no labeling was observed for MtrA at any incubation duration (Figure 1B, panel i), indicating that AcP serves as an acetyl donor, but not a phosphoryl group donor for MtrA. As a control, phosphorylation of MtrA from MtrB was recorded as reported previously (Figure 1B, panel ii) (Agrawal et al., 2015; Singh et al., 2019). Previously, Lys^110^ was identified as an acetylation site on MtrA when acetylation was enzymatically catalyzed (Singh et al., 2019). We probed for the acetylation site in the MtrA protein after AcP treatment using ESI MS/MS analysis. We identified three lysine residues at positions 108 (Figures 1C vs. D and Supplementary Figure 1C), 110 (Figures 1C vs. E and Supplementary Figure 1D), and 207 (Figures 1F vs. G and Supplementary Figure 1E) to be acetylated. This demonstrated that, irrespective of the mechanism of reaction catalysis viz. enzymatic or non-enzymatic, the same lysine residue K110 is acetylated in the RR MtrA. However, non-enzymatic acetylation occurs on a larger number of lysine residues in comparison to enzymatically catalyzed reactions.

### Acetylation of RR MtrA Alters Its Interaction Affinity With SK, MtrB

**FIGURE 2:**
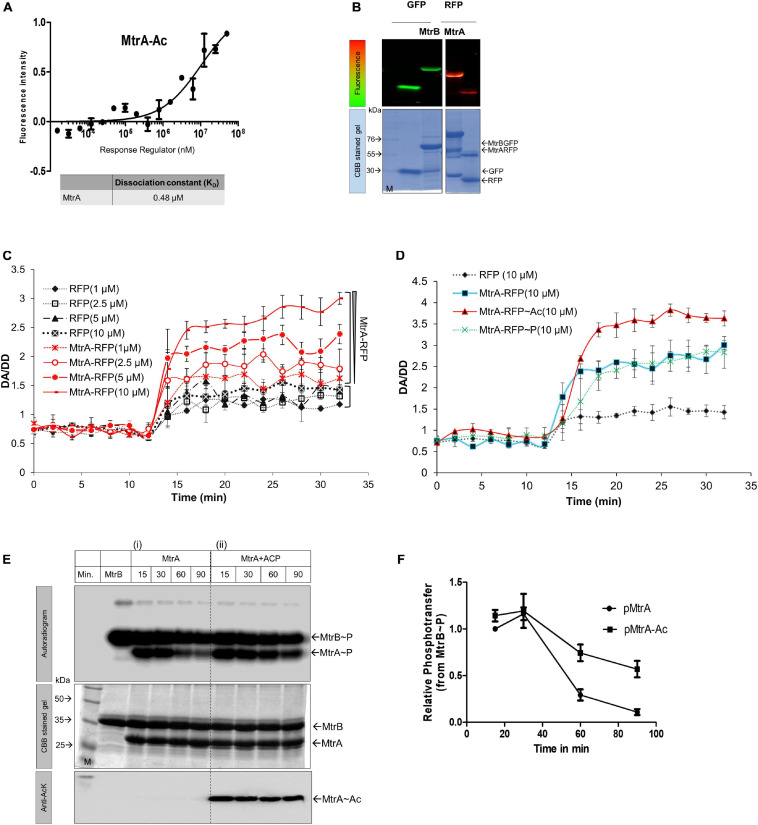
Interaction analysis of MtrA. **(A)** Analysis of MtrA interaction with AcP. MST measurements for determining interaction affinity of AcP with MtrA (*n* = 3). **(B)** SDS-PAGE analysis of purified fluorescently tagged fusion proteins. The top panels represent the fluorescence gel image of various tagged proteins used for FRET studies. Left, GFP and MtrB-GFP; right, MtrA-RFP and RFP. Bottom panels, corresponding CBB stained gels. **(C)** Effect of acetylation on the interaction between MtrB-GFP and MtrB-RFP (solid lines) or RFP alone (dotted lines) performed at various concentrations of acceptor molecule (1–10 μM) by FRET. RFP protein alone was used as a negative control. FRET analysis was performed by measuring FRET (DA [donor excitation and acceptor emission]/DD [donor excitation donor emission]. **(D)** Effect of acetylation and phosphorylation on the interaction between MtrB-GFP and MtrA-RFP as measured by FRET. RFP protein alone was used as a negative control. **(E)** Phosphotransfer time course to analyze the effect of acetylation on MtrA phosphorylation from its cognate SK, MtrB. The assay was performed using (i) wild-type MtrA, (ii) acetylated MtrA (with AcP). (M, marker). 1st panel, autoradiogram; 2nd panel, CBB stained gel; and 3rd panel, the image of blot probed for acetylated lysine. **(F)** Quantitative measurement of MtrA phosphorylation through sensor kinase, MtrB at various time points (data from experiments reported in **E**). Signal recorded for unacetylated MtrA at 5-min time point taken as 1, and the rest were normalized to that.

We observed that the acetylated MtrA is very stable, thereby allowing us to extensively investigate its effect on the various activities of the RR. Different reactions in which RRs typically participate were used to study the impact of acetylation. These include (i) RR interaction with SK, phosphotransfer reaction from SK, RR binding to the target DNA, and dephosphorylation of the RR itself. The RR protein’s first activity involves interaction with SK, which is necessary for the phosphotransfer reaction. Here, the phosphoryl moiety from the activated SK MtrB is transferred to the RR MtrA. Previously, it has been shown that enzymatically acetylated MtrA is a better phosphoacceptor from MtrB (Singh et al., 2019). To determine the binding affinity of AcP to the MtrA protein, we generated a fluorescently (RFP) tagged MtrA protein for MST analysis, similar to what has been reported previously (Winiewska et al., 2017; Singh et al., 2019). The fluorescently tagged protein was first confirmed to be active in the phosphotransfer assay from MtrB (Supplementary Figures 2A,B). Using the tagged protein, MST measurements yielded an interaction affinity of 0.48 μM for AcP to MtrA (Figure 2A). Interestingly, the maximum physiological concentration of AcP in a cell has been measured to range between 100 μM and 3 mM, including in *M. tuberculosis* (Table 1) (Zhao et al., 2005; Klein et al., 2007; Ramos-Montañez et al., 2010; Sharma et al., 2019), making the interaction physiologically relevant. Interestingly, the CD spectroscopy analysis displays no difference in the secondary structure of the acetylated MtrA protein when compared with non-acetylated MtrA (Supplementary Figure 2C).

**TABLE 1:** The physiological concentration of AcP reported in several prokaryotes.

**Species**	**Physiological concentration of AcP**	**References**
*Escherichia coli*	100 μM–3 mM	Wolfe, 2005; Klein et al., 2007
*Streptococcus pneumoniae*	3 mM	Ramos-Montañez et al., 2010
*Clostridium acetobutylicum*	143 pmol/g	Zhao et al., 2005
*Mycobacterium tuberculosis*	3.7 mM	Sharma et al., 2019

Next, we examined if acetylation altered SK-RR interaction affinity using a FRET-based interaction analysis approach. For this, GFP-tagged MtrB and RFP-tagged MtrA proteins were generated and used (Figure 2B), and they were confirmed to be active similar to the untagged protein in an optimized radioactive phosphorylation assay (Saini et al., 2004) (Supplementary Figures 2A,B). A FRET-based assay was used previously to study the interaction between SK and RR proteins (Agrawal et al., 2016). Basal FRET indicative of steady-state interaction between GFP-MtrB and RFP-MtrA proteins was evaluated at first. This was performed by analyzing FRET in the presence of increasing amounts of the acceptor, either RFP (negative control) or MtrA-RFP (test protein) with a fixed concentration of donor MtrB-GFP (Figure 2C). The FRET efficiency plotted as a change in DA/DD (GFP excitation RFP emission/GFP excitation GFP emission) as a function of time showed low FRET when just RFP was used as the acceptor molecule. This was due to the inherent property of fluorescent proteins to dimerize at a low rate (Zacharias et al., 2002), and it did not change when the number of RFP molecules in the reaction was increased (Figure 2C). In the presence of MtrA-RFP, the FRET was higher than RFP alone. The increase was commensurate with the amount of donor in the reaction, and maximum FRET was recorded in the presence of 10 μM of MtrA-RFP (Figure 2C), suggestive of an interaction between SK, MtrB and RR, MtrA. Using this assay, changes in the interaction were probed between MtrA with MtrB in the presence of acetylation or phosphorylation. RFP alone was used as a baseline. Although interaction of MtrA or MtrA∼P with MtrB was almost similar, a higher interaction was recorded when MtrA was acetylated using AcP (Figure 2D). Overall, it was observed that SK MtrB and RR MtrA interact with each other under steady-state conditions, and their interaction is enhanced when MtrA is acetylated but not altered by phosphorylation.

### Acetylated MtrA Is Dephosphorylated by SK MtrB at a Lower Rate

Next, we probed if the enhanced interaction between the SK and RR proteins, modulated by non-enzymatic acetylation, could impinge on the phosphotransfer efficiency from MtrB to MtrA protein. Toward this, a time-course analysis of the MtrB to MtrA phosphotransfer reaction was performed in conditions in which MtrA was either non-acetylated or acetylated using AcP. We observed that the acetyl group on MtrA enhanced retention of phosphoryl moiety from MtrB compared to non-acetylated MtrA (Figure 2E, panel i vs. ii). This was especially conspicuous at later time points and was quantitatively different (Figure 2F), similar to what has been observed previously through the enzymatic acetylation (Singh et al., 2019). This observation suggests that acetylation of MtrA hinders the MtrB phosphatase activity rather than the phosphor-acceptance ability. The phosphotransfer analysis also revealed higher stability of Ac-MtrA∼P, which could translate into the higher or prolonged transcriptional output from the RR MtrA. The impact of this observation is discussed in the following sections. We also tested if AcP can acetylate SK MtrB, and it was absent (not shown), thereby confirming that the observed effects are only due to acetylation of MtrA.

### Differential Effects of Acetylation and Phosphorylation on the DNA-Binding Activity of MtrA

Given that MtrA is an RR protein that functions as a transcription factor by directly interacting with specific DNA targets (Zahrt and Deretic, 2001; Bretl et al., 2011; Purushotham et al., 2015; Sharma et al., 2015), we then tested if its DNA binding abilities are altered as a result of acetylation. Toward this, we examined binding on two distinct targets of MtrA, *oriC*, and *rpfB* (Purushotham et al., 2015; Sharma et al., 2015). MtrA has been reported to bind to *oriC*, where it acts as a repressor of replication (Purushotham et al., 2015). Similarly, it prevents reactivation of latent bacilli by binding to the *rpfB* promoter region (Sharma et al., 2015).

**FIGURE 3:**
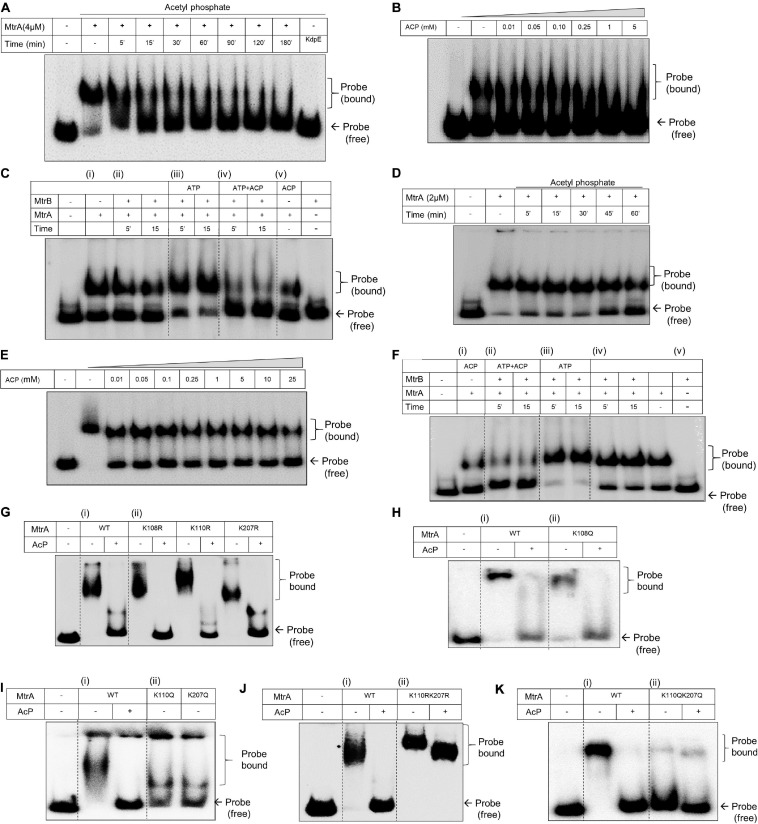
Analysis of DNA binding activity of MtrA by electrophoretic mobility shift assay (EMSA). **(A)** Analysis of MtrA binding on *oriC* DNA as a function of acetylation mediated through AcP (5 mM) for the indicated time durations. RR KdpE was used as a negative control. Corresponding acetylation time course of MtrA is shown in Supplementary Figure 4A. **(B)** Titration of AcP concentration sufficient for complete acetylation of MtrA and inhibiting its DNA binding activity. MtrA protein was incubated with an increasing concentration of AcP (as specified) and tested for changes in its DNA binding on *oriC*. **(C)** Comparative evaluation of phosphorylation and acetylation on the binding of MtrA on the *oriC* DNA. Wild-type MtrA was phosphorylated using MtrB∼P or acetylated (by AcP) and incubated with *oriC* promoter DNA. MtrB (lane 10) was used as a negative control to determine non-specific binding with *oriC* DNA region. **(D)** Analysis of MtrA binding on *rpf* promoter region as a function of acetylation mediated through AcP (5 mM) for indicated time durations. **(E)** Titration of AcP concentration sufficient for complete acetylation of MtrA and inhibiting its DNA binding activity. MtrA protein was incubated with an increasing concentration of AcP (as specified) and tested for changes in its DNA binding on *rpf*. The corresponding acetylation time course of MtrA is shown in Supplementary Figure 4B. **(F)** Comparative evaluation of phosphorylation and acetylation on the binding of MtrA on the *rpfB* promoter DNA. Wild-type MtrA was (i) acetylated (by ACP); (ii) phosphorylated using MtrB∼P and acetylated by AcP; (iii) only phosphorylated using MtrB∼P and incubated with *rpf* promoter DNA as per the conditions mentioned in each lane. Panel (iv) control with AcP and phosphorylation and (v) MtrB used as a negative control to determine non-specific binding with *rpf* promoter. **(G–K)** Analysis of MtrA binding on the *rpf* promoter region for (i) wild type and (ii) various Lys mutants of MtrA proteins (as indicated). The binding of proteins was also analyzed as a function of acetylation through AcP (5 mM) as shown. KR substitution generates an acetylation defective mutant, and KQ substitution generates acetylation mimic mutant protein. The bound probe marks the shift.

For analysis of binding of MtrA protein on the DNA fragment corresponding to the 526 bp *oriC* DNA fragment, an initial titration with increasing concentrations of purified protein was performed. Here, a robust binding of MtrA on the DNA revealed by electrophoretic mobility shift was observed (Supplementary Figure 3A). When the effect of AcP was examined on MtrA (4 μM) binding to the DNA, it was observed that the presence of AcP (5 mM) reduced its DNA binding ability as a function of incubation time (Figure 3A). With this observation, we next titrated the concentration of AcP, which prevented the binding of MtrA on the *oriC* DNA region. A dose of 10 μM to 5 mM was tested, and a reduction in DNA binding ability as a function of concentration was observed within 30 min (Figure 3B). Next, the impact of SK-dependent phosphorylation on the MtrA DNA binding property was examined. Although the presence of MtrB *per se* did not affect the DNA binding ability of MtrA (Figure 3C, panel i vs. ii), phosphorylation of MtrA through MtrB enhanced the DNA binding ability (Figure 3C, panel ii vs iii). Interestingly, acetylation is the dominant one of the two post-translational modifications, and irrespective of its phosphorylation status, acetylated MtrA binding was lower to DNA (Figure 3C, panel iv) compared with unmodified wild-type or phosphorylated MtrA protein.

This phenomenon was probed for another target *rpfB*, and similar observations were made. Although MtrA (2 μM) bound to the DNA strongly (Figure 3D and Supplementary Figure 3B), its DNA binding ability was suppressed in the presence of AcP as a function of time (Figure 3D) and as a function of the concentration of AcP (Figure 3E). Phosphorylation enhanced the DNA binding activity to the *rpfB* promoter region (Figure 3F, panel iii), which was also suppressed when acetylated MtrA was used in the reaction (Figure 3F, panel ii vs. iii).

To determine acetylation of which specific lysine residue (from 108, 110, and 207) affects the DNA binding activity, we generated acetylation defective (KR substitution) and acetylation mimic (KQ substitution) mutant proteins of MtrA. These mutant proteins were tested by EMSA for binding to the *rpfB* promoter and *oriC* DNA region in both the presence or absence of AcP. EMSA analysis of all the acetylation-defective mutant proteins viz. MtrAK108R, MtrAK110R, and MtrAK207R showed similar binding properties to the *rpfB* promoter (Figure 3G) and *oriC* region (Supplementary Figure 3C) as the wild-type MtrA. Interestingly, although no changes for acetylation mimic mutant MtrA K108Q were recorded on either DNA (Figure 3H and Supplementary Figure 3D) for MtrA K110Q and MtrA K207Q proteins, reduced binding to the *rpfB* promoter (Figure 3I) as well as on the *oriC* region (Supplementary Figure 3E) was recorded. This suggested that both Lys^110^ and Lys^207^ play an important role in regulating the DNA binding activity of MtrA (Figure 3I).

To further extend these observations, we generated double mutants of MtrA (both acetylation defective and mimic) viz. MtrA K110R K207R and MtrA K110Q K207Q and analyzed their DNA binding activity. Confirming our hypothesis, acetylation-defective double mutant protein (KR substitutions), bound *rpfB* promoter and *oriC* DNA similar to the wild-type MtrA protein (Figure 3J and Supplementary Figure 3F). There was no impact of acetylation for the defective protein (through AcP), which could abolish DNA binding of wild-type protein (Figure 3J and Supplementary Figure 3F, last lane). In agreement with this, the acetylation mimic (KQ) MtrA completely failed to bind to the *rpfB* promoter and *oriC* promoter DNA, independent of the presence of AcP (Figure 3K and Supplementary Figure 3G). Overall, we show that Lys^110^ and Lys^207^ regulate the DNA binding ability of MtrA and tune it through AcP dependent acetylation.

### RR MtrA Can Use AcP as an Acetyl Donor *in vivo*

**FIGURE 4:**
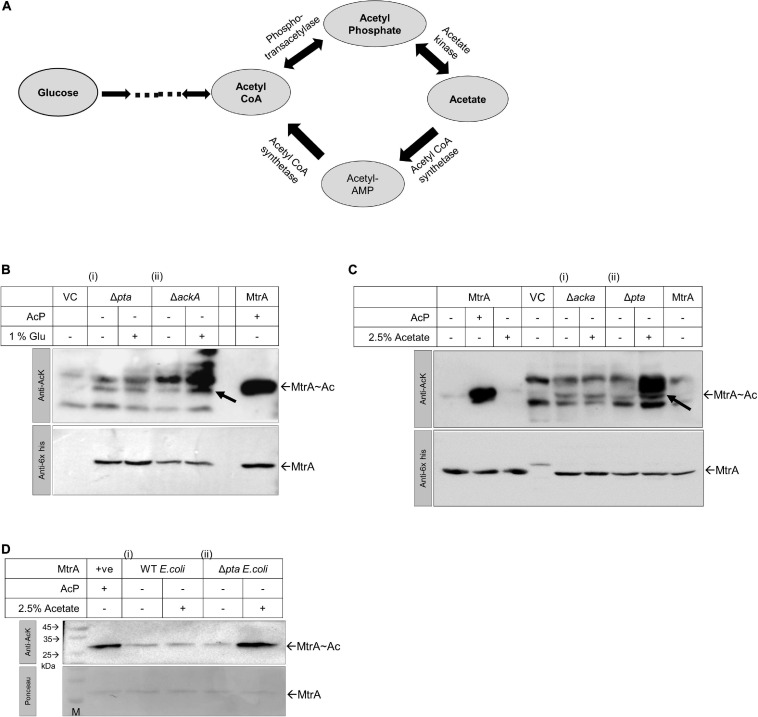
Analysis of MtrA acetylation *in vivo* in *E. coli* using Δ*ackA* and Δ*pta* knockout strains. **(A)** Biochemical reaction schemes through which AcP is generated *in vivo* from glucose, acetyl CoA, or acetate. Enzymes engaged in the reaction schemes involving changes in the levels of AcP are indicated. **(B)** Acetylation status analysis for MtrA protein expressed in *E. coli* BL21 (i) Δ*pta* and (ii) Δ*ackA* strains in the presence or absence of 1% glucose supplemented Luria broth medium (LB) by Western blotting (purified MtrA acetylated protein used as positive control and VC, pPROEx vector alone). **(C)** Acetylation status analysis for MtrA protein expressed in *E. coli* BL21 (i) Δ*ackA* and (ii) Δ*pta* strains grown in the presence of 2.5% acetate supplemented LB by Western blotting (+ ve control, purified MtrA acetylated protein and VC, pPROEx vector alone). **(D)** MtrA proteins purified from *E.coli* BL21 (i) wild-type strain and (ii) Δ*pta* strain, which were further verified for their acetylation status. For panels **(B–D)**, top, blot probed with anti-acetyl lysine antibody and, bottom with an anti-6x-his antibody for normalization (*n* = 3).

To test if RR MtrA can accept the acetyl group from AcP *in vivo*, we probed for the presence of acetylation on MtrA protein purified from *E. coli*, where this protein was overexpressed. We modulated the pool of AcP in *E. coli* cells using *pta* and *ackA* mutant strains and then probed for acetylation in the cells vis-à-vis total MtrA expressed in them. *In vivo*, AcP is generated from acetyl CoA through the activity of enzyme phospho transacetylase (*pta*) or from acetate through the enzyme acetate kinase (*ackA*). The ackA enzyme also catalyzes the interconversion of AcP to acetate, which also feeds into the acetyl-CoA pool (Figure 4A).

When *E. coli* cells are grown in the presence of glucose, the intracellular level of AcP is higher in the Δ*ackA* strain as in the absence of acetate kinase (AckA), AcP is not converted to acetate. But when cells are grown in acetate containing media, the AcP levels are higher in the Δ*pta* strain (Wolfe, 2005) as here the AcP is not converted to acetyl CoA (Figure 4A). Using these strains, we expressed MtrA and tested for *in vivo* acetylation, and higher acetylation in the Δ*ackA* strain was recorded in the presence of glucose by anti-acetyl Western blotting (Figure 4B, panel ii). This was true not only for overexpressed MtrA, but also for other cellular proteins of *E. coli* as well (Figure 4B, top panel), similar to what was observed previously (Kuhn et al., 2014). The MtrA acetylation was normalized with reference to expression levels of the MtrA protein itself using an anti-his antibody (Figure 4B, bottom panel). When the same experiment was performed in the presence of acetate, as anticipated, higher acetylation was recorded in the Δ*pta* strain for both overexpressed MtrA protein and other *E. coli* proteins (Figure 4C, panel ii vs. i).

Given that acetylated MtrA protein is stable, we further analyzed the presence of *in vivo* acetylation by purifying the proteins from mutant strains grown in the presence of 2.5% acetate. As anticipated, higher acetylation on the MtrA protein was recorded in protein purified from the Δ*pta* strain grown in the presence of acetate (Figure 4D, panel ii vs. i). Our studies thereby confirm that MtrA could accept the acetyl group from AcP under *in vivo* conditions, potentially altering its molecular functions described in the sections above.

### Lysine Acetylation of MtrA Affects the Growth and Pathogenesis of *M. tuberculosis* H37Ra

**FIGURE 5:**
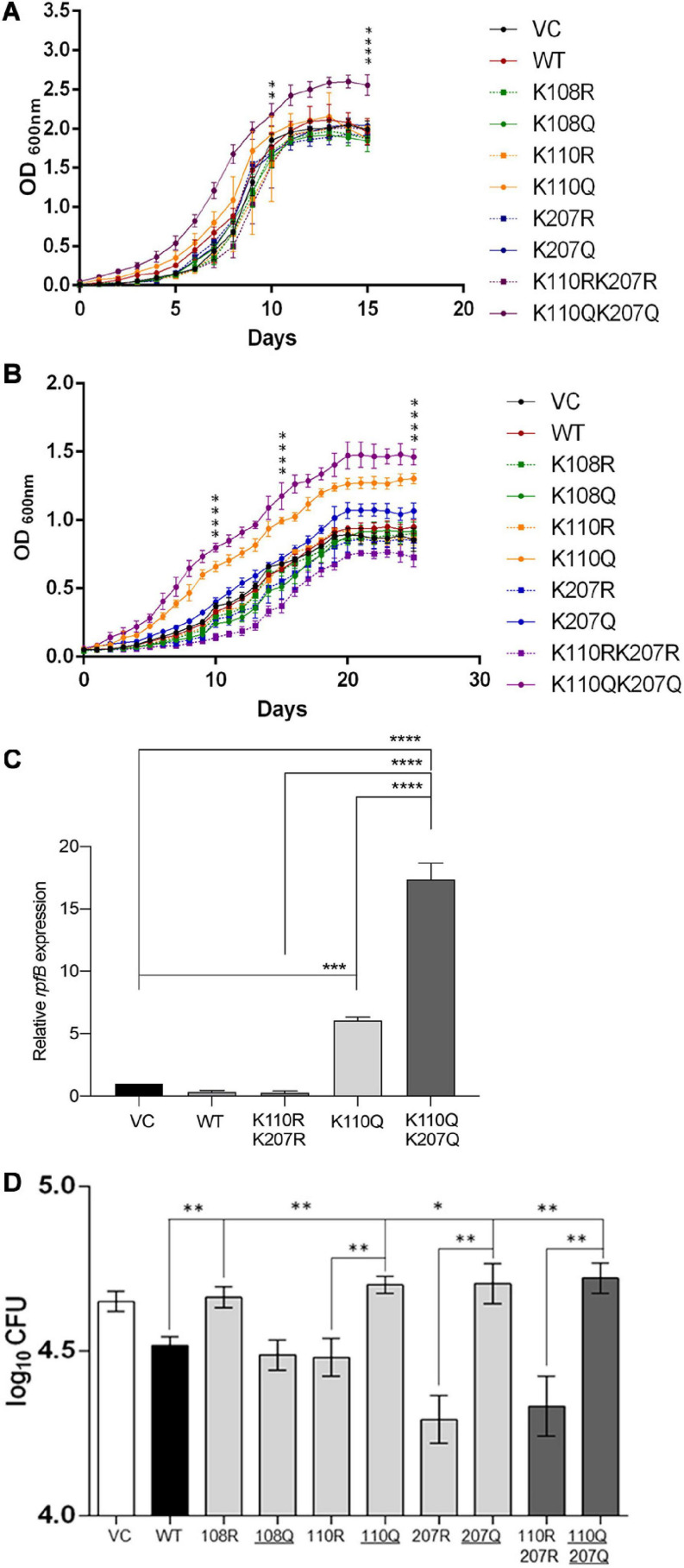
Analysis of growth and invasion of *M. tuberculosis* H37Ra merodiploid strains carrying various lysine mutations in the MtrA protein. **(A)** Comparison of growth of various strains (as indicated) in nutritionally rich media. The OD_600_ of cultures were recorded every day and plotted as a function of time (days). The significance (*p* values) was calculated on days 10 and 15. **(B)** Comparison of growth of various strains (as indicated) in low-nutrition media (7H9 alone). The OD_600_ of cultures were recorded every day and plotted as a function of time (days). The significance (*p* values) was calculated on days 10, 15, and 25. **(C)** Analysis of *rpfB* expression in the experiment described in panel B by quantitative RT-PCR. **(D)** A549 alveolar epithelial cells were infected with various H37Ra strains containing pMV261 plasmid with various variants of MtrA (as indicated) (VC; pMV261 vector alone), following which CFU was determined at 4 hpi. Log_10_ CFU were further calculated for each strain and compared with each other. KQ mutations that generate acetylation mimic variant are underlined. For all graphs, significance (*p*-value) was calculated by student’s *t*-test and is represented as *, where * ≤ 0.05, ** ≤ 0.01, *** ≤ 0.001, and **** ≤ 0.0001.

To evaluate the physiological impact of lysine acetylation in MtrA proteins, we generated merodiploid strains overexpressing MtrA protein carrying various lysine mutants (both acetylation defective or acetylation mimic as described above). Given that MtrAB TCS and, more specifically, RR MtrA regulate the cell division and growth in *Mycobacterium* spp. (Fol et al., 2006; Al Zayer et al., 2011; Purushotham et al., 2015; Kundu, 2018), we initially examined the impact of acetylation variants of MtrA on growth in nutrient-rich and nutrient-deficient media. We observed that, in nutrient-rich media, all the MtrA mutants grow similarly to the strain carrying wild-type MtrA protein except for the strain carrying the double mutant of lysine acetylation mimic, MtrA K110Q K207Q (Figure 5A). This observation suggests that acetylation on these lysines, which abolishes the DNA binding activity of MtrA (Figure 3K), removes the repressor activity of MtrA, and increases the cell proliferation rate as reported previously (Purushotham et al., 2015).

Furthermore, in nutrient-deficient media, both single acetylated lysine mimic MtrA K110Q as well as double lysine mimic mutant MtrA K110QK207Q (Figure 5B) strains grow better. This suggests that acetylation at Lys^110^ serves as an AcP-sensitive acetylation site, which affects the growth of mycobacterial cells. Given that the AcP pool is higher in nutrition-rich media (Wolfe, 2005; Schilling et al., 2015), the impact of Lys^110^ acetylation is more evident in nutrient-poor media, in which all strains grow equally, and the role of acetylation is minimal. This observation was also examined at the molecular level, where the change in the expression of *rpfB*, a target gene whose expression is repressed by MtrA (Fol et al., 2006), was examined in various cultures grown in poor media for 15 days. Similar to what was recorded in growth experiments, the levels of *rpfB* were higher in strains carrying acetylation mimicking mutation at the Lys^110^ position (MtrA K110Q). The enhanced expression was significantly higher (16-fold) in the strain carrying the MtrA K110QK207Q double mutant (Figure 5C).

Further, to analyze the impact of acetylation of MtrA on the pathogenesis of *M. tuberculosis in vivo*, infection in A549 cells, an alveolar epithelial cell line, was performed. Host cells were infected with various strains (as mentioned above) at an moi of 10, and cfu were recorded at 4 and 24 hpi. Given that overexpression of MtrA wild type reduces the infectivity of *M. tuberculosis* (Fol et al., 2006), in our experiments, we also observed lower cfu in the MtrA-expressing strain compared with the strain containing the vector alone (Figure 5D). In the strains with acetylation defective MtrA 207R and its double mutant K110R K207R, a lower bacterial load was observed (Figure 5D), which was reversed, and a high load was recorded when acetylation-mimicking mutations were present (Figure 5D). Interestingly, an acetylation-defective lysine mutation at position 108 (MtrA K108R) reversed the MtrA overexpression effect, resulting in a reduced bacterial load inside A549 cells (Figure 5D), which was lost when the acetylation mimic mutation was introduced. These findings reveal many exciting aspects of MtrA acetylation in the infection process of *M. tuberculosis*. First, acetylation of MtrA at Lys^108^ is essential for its repressive activity, which regulates the proliferation rate as reported previously (Fol et al., 2006) and confirmed by a reduced bacterial load in the merodiploid strain and loss of reduction in the Lys^108^ defective strain. Unlike this, acetylation of Lys^110^ and Lys^207^ is critical for its derepression and consequent enhancement of MtrA-mediated activity viz. cell proliferation, which also revealed *in vitro* growth analysis (Figures 5A–C). Although the role of Lys^110^ acetylation is more critical for regulating the *in vitro* growth rate, acetylation of both the Lys^110^ and Lys^207^ seems vital for controlling the growth rate *in vivo* in the host cell. The same findings were recapitulated at the 24-hpi time point (Supplementary Figure 5).

**FIGURE 6:**
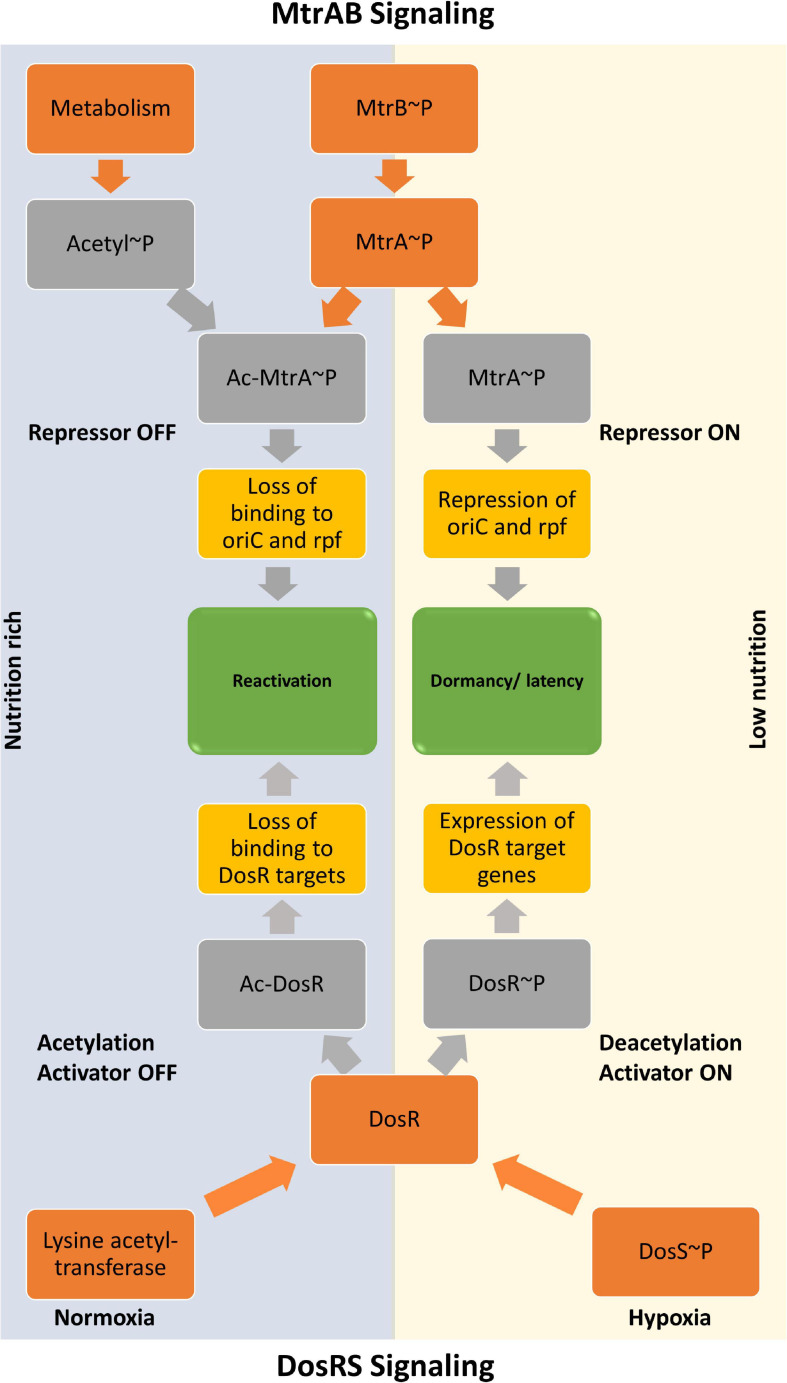
Model showing regulation of activities of MtrA response regulator by acetylation and phosphorylation. The input driven by a stimulus that activates sensor kinase MtrB is dominated by the metabolic cues generated inside the cell, which is sensed through the acetylation process. The regulatory procedures allow the MtrAB system to regulate the resuscitation program in response to an increased acetylation pool, which, at the same time, turns OFF the DevRS mediated dormancy program.

Overall, we demonstrate that the acetylation of RR MtrA significantly alters its behavior as a regulatory molecule and establishes alternate signaling landscapes depending on post-translational modification on the RR. MtrA regulons can be acetylation- or phosphorylation-dependent because of the concerted action of both acetylation and phosphorylation. We also present the dominant effect of acetylation-dependent signaling on regulating the RR MtrA, which overrides phosphorylation. Our studies thereby suggest a model (Figure 6), in which the nutritional sensing, through non-enzymatic acetylation (Schilling et al., 2015), dominates the extracellular stimulus transmitted through SK MtrB. The design also permits a dispensable role for MtrB as proposed and an essential repressor role for MtrA. Our experiments also reveal that, although acetylation and phosphorylation regulate two different activities of the MtrA protein, the effect of acetylation is dominant.

## Discussion

In a typical two-component signaling cascade, phosphorylation-mediated gene expression changes are the most well-characterized adaptive response associated with them. It is by far the most widely studied adaptive response for TCS, primarily on account of changes recorded in the DNA binding activity of the RR proteins through changes in their phosphorylation status (Stock et al., 2000; Gao et al., 2007; Gao and Stock, 2009). Over the years, similar to the eukaryotic regulator proteins, in which acetylation-dependent gene expression modulation is more prevalent (Verdin and Ott, 2015), the same has been recorded for RR proteins (Christensen et al., 2019a). Given that acetylation is a reversible PTM, it is as attractive as phosphorylation for regulating activities of transcription factors (Kim and Yang, 2011; Thao et al., 2011; van Noort et al., 2012).

It has recently been shown that hypoxia regulon of RR DosR, which is modulated by phosphorylation (Saini et al., 2004; Sharma et al., 2019), is also regulated by its acetylation (Bi et al., 2018; Yang et al., 2018). The study proposes that acetylation is reduced during hypoxia, which increases its DNA binding ability and thereby turns on the dormancy regulon (Bi et al., 2018; Yang et al., 2018). These findings are in sync with the work presented here, in which (i) reduced DNA binding for MtrA is recorded when it is acetylated, and (ii) a regulatory effect of acetylation on the tuning growth of mycobacteria through acetylation of MtrA protein is demonstrated in both *in vitro* and *in vivo* conditions. The second outcome supports prior evidence in which regulation of mycobacterial cell division is controlled by MtrA (Purushotham et al., 2015; Sharma et al., 2015), thus providing a role for non-enzymatic acetylation of MtrA in regulating mycobacterial resuscitation and proliferation, driving reactivation of *M. tuberculosis* from dormancy, in which MtrA operates like a repressor as proposed previously (Sharma et al., 2015). Interestingly, in a recent study, the deletion of MtrB affected virulence of *M. tuberculosis* and primarily impaired the bacilli’s ability to form granuloma (Banerjee et al., 2019). The study also showed that the DosRS regulon is downregulated in the absence of MtrB, and it happens due to the direct interaction of SK MtrB with RR DosR (Banerjee et al., 2019). However, they have not discussed the impact of phosphorylation or acetylation on the activities of any RR, either RRs, MtrA, or DosR.

The dominant effect of acetylation of MtrA is also in agreement with previous reports, which show that mycobacteria can tolerate the deletion of the *mtrB* gene but not of *mtrA* gene (Zahrt and Deretic, 2000; Möker et al., 2004; Plocinska et al., 2012; DeJesus et al., 2017). Also, the regulatory role of MtrA is phosphorylation independent (Plocinska et al., 2014). It has been proposed that resuscitation of dormant *Mtb* is triggered by the expression or presence of *rpf* (Mukamolova et al., 2010) or environment, which is more conducive for the growth of bacterial cells, which would include the availability of high carbon sources. Given that acetylation suppresses DosR activity and promotes activation of *oriC* through MtrA, it is plausible to speculate that the presence of a carbon-rich environment fosters the proliferation of the tubercle bacilli and suppresses the dormancy response of *M. tuberculosis*. This nutrient availability–dependent reactivation of dormancy bacilli could be a straightforward but elegant approach that agrees with the dynamic model of latent TB infection (LTBI) proposed previously (Hampshire et al., 2004; Voskuil, 2004; Dutta and Karakousis, 2014; Gibson et al., 2018; Menzies et al., 2018). We show here that these regulatory proteins can directly modulate their responses through metabolic molecules, such as AcP without involving any enzymes. The repressor activity of MtrA is also in agreement with the higher binding of the acetylated MtrA with MtrB as it also keeps the protein together and ready for switching immediately if needed. The study model is presented in Figure 6.

## Data Availability Statement

The datasets generated for this study are available on request to the corresponding author.

## Author Contributions

KS designed the study, performed the experiments, analyzed the data, and wrote the manuscript. PA performed EMSA, *in vivo* acetylation, growth curve, and infection experiments. NB performed the MS/MS studies. DS performed the MST experiments. UW performed CD and RT-PCR experiments and analyzed the data. DS conceived the study, analyzed the data, and wrote the manuscript. All authors contributed to the article and approved the submitted version.

## Conflict of Interest

The authors declare that the research was conducted in the absence of any commercial or financial relationships that could be construed as a potential conflict of interest.
