# Supplementary material for: Acetylation of Response Regulator Protein MtrA in M. tuberculosis Regulates Its Repressor Activity
Source: Front Microbiol. 2021 Jan 15;11:516315. doi: 10.3389/fmicb.2020.516315 (PMC7843721; doi:10.3389/fmicb.2020.516315)
Supplement: Supplementary file 1 [file Data_Sheet_1.pdf]

Supplementary Figure 1.

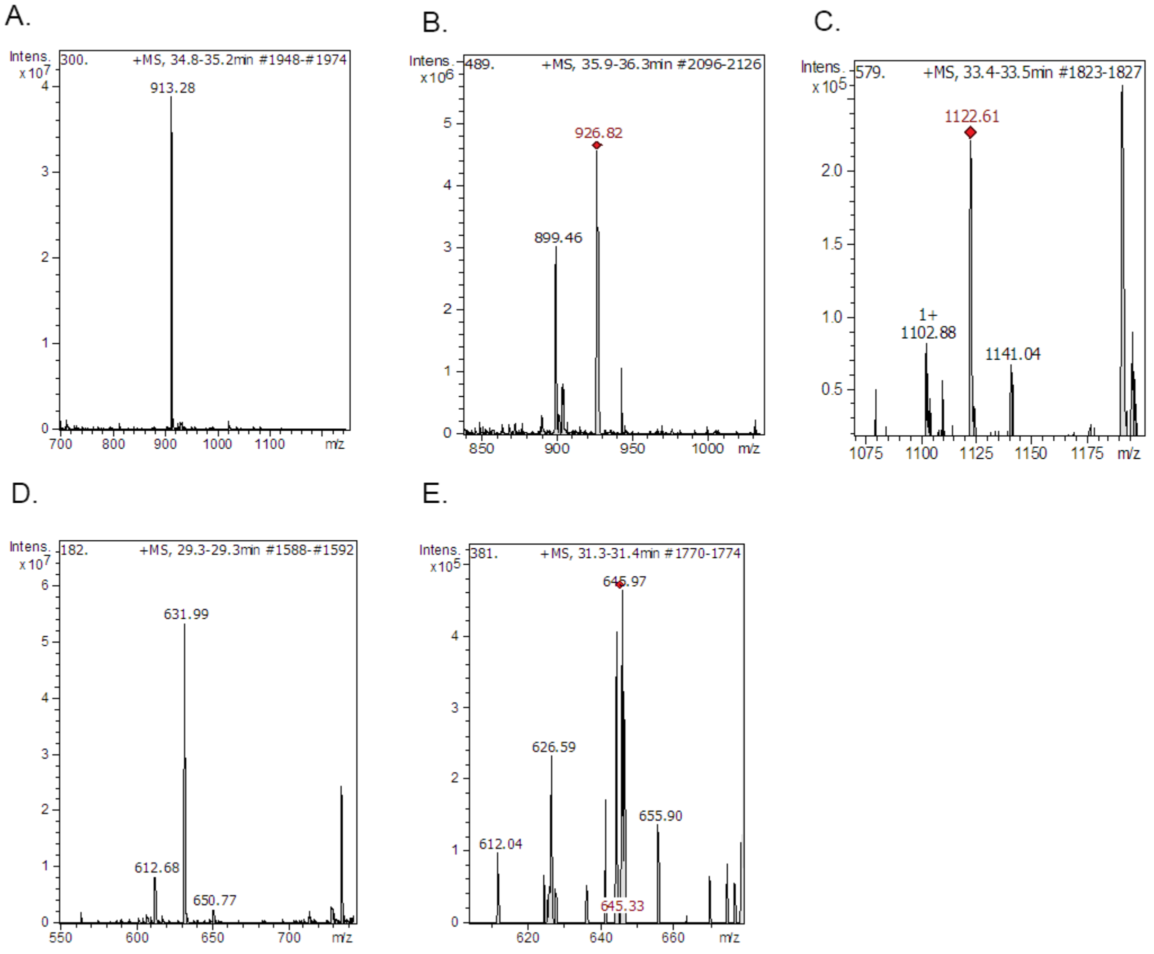

Supplementary Figure 1. A-E. **Identification of lysines acetylated in MtrA protein through AcP by MS/MS analysis**. Precursor ion spectra for **A.** MtrA-K108; **B.** MtrA-K110; **C.** MtrA-K108 acetylated; **D.** MtrA-K110 acetylated and **E.** MtrA-K207 acetylated. For all MS analysis, n=3 from independent samples.

Supplementary Figure 2.

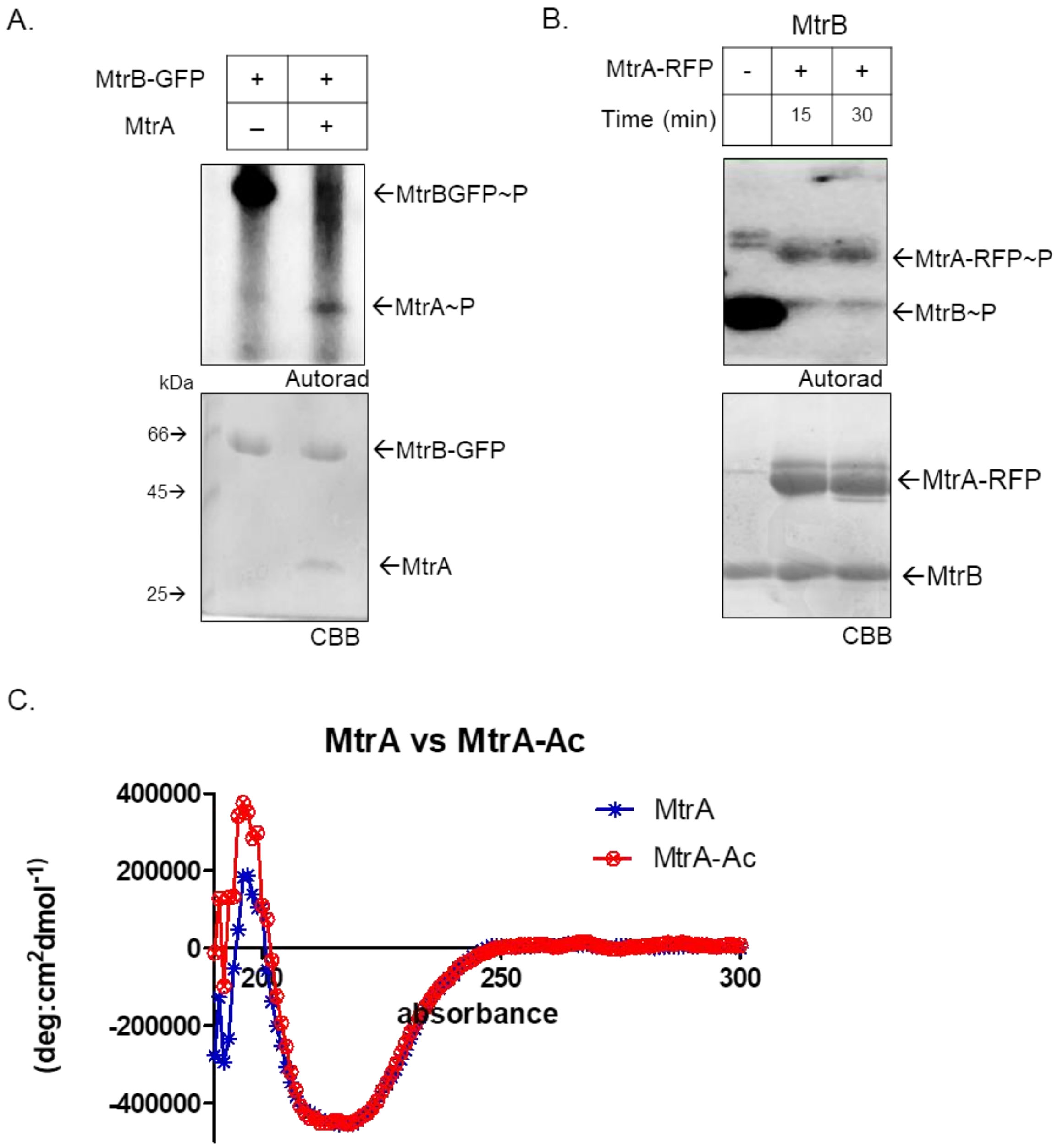

Supplementary Figure 2. A. Autophosphorylation and phosphotransfer analysis of GFP-tagged MtrB protein to MtrA. Top, autoradiogram; bottom, CBB stained gel. **B.** Autophosphorylation and phosphotransfer analysis of MtrB protein to RFP tagged MtrA protein. Top, autoradiogram; bottom, CBB stained gel. All the experiments were performed as described in the materials and methods section**. C.** Circular dichroism analysis of MtrA and MtrA with AcP proteins. MtrA proteins were evaluated using a Jasco Spectropolarimeter. Ellipticity was measured from 200 nm to 300 nm in 1 mm pathlength and 50nm/ sec scanning speed. The data were analyzed through K2D2 Dichroweb software.

Supplementary Figure 3.

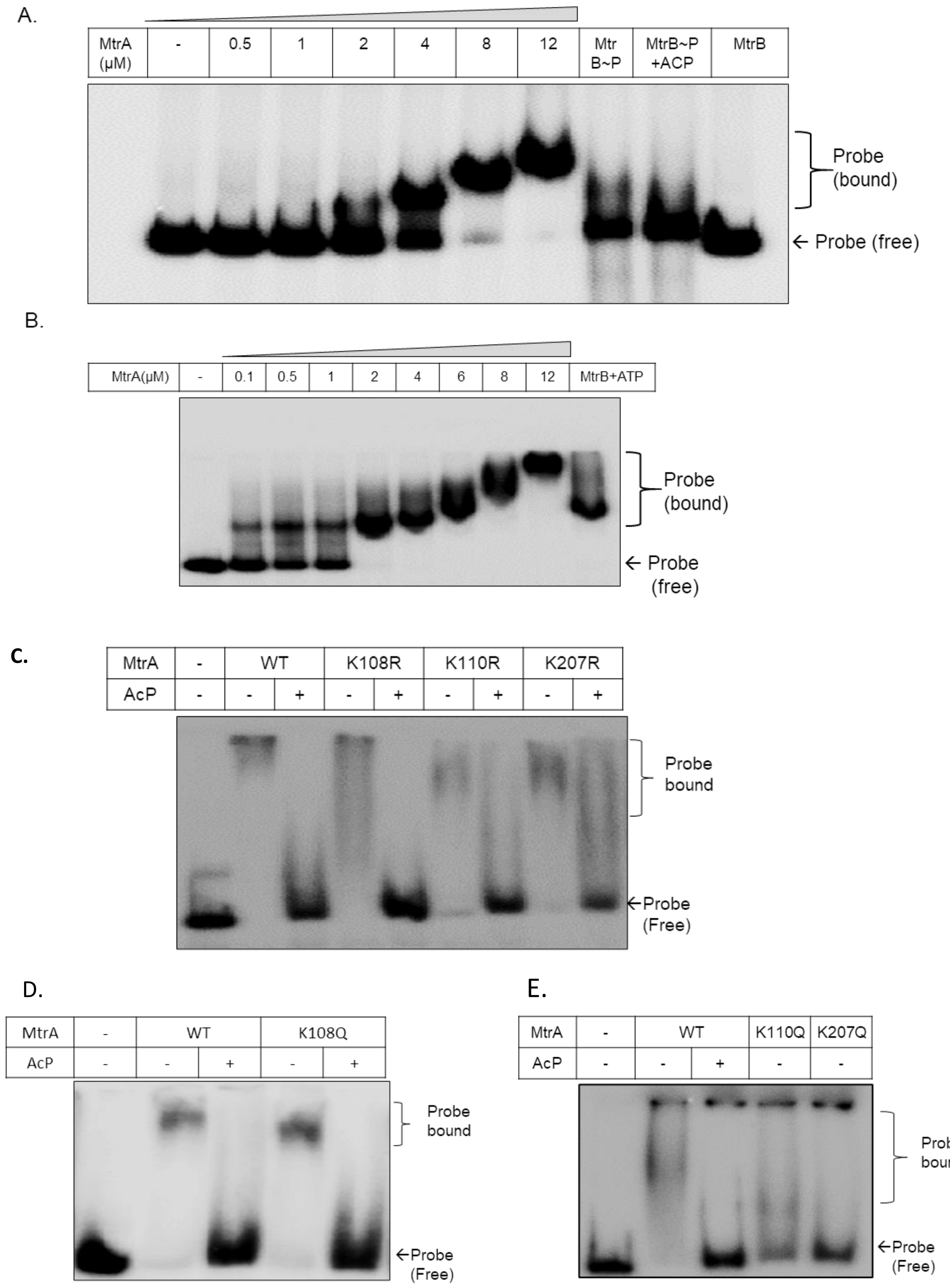

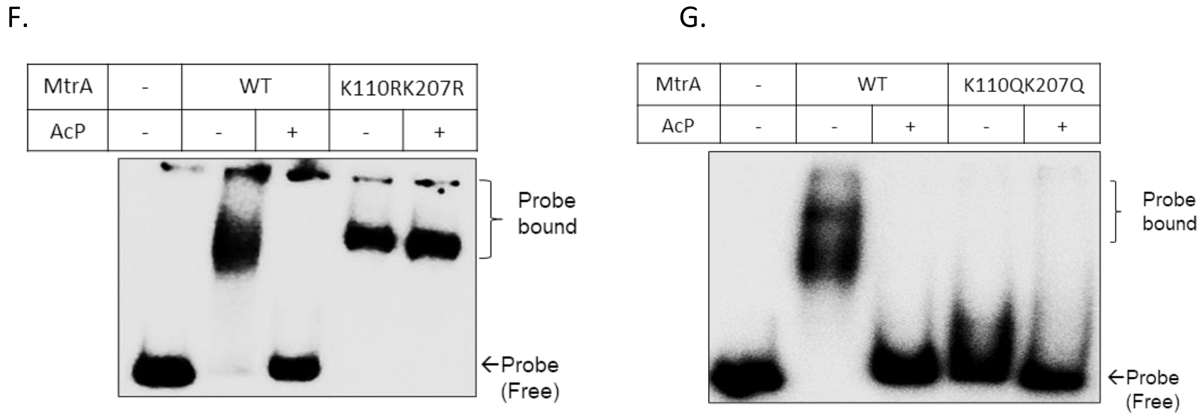

Supplementary Figure 3. Electrophoretic mobility shift assay (EMSA) with *oriC* promoter and *rpfB* promoter for dose titration on DNA binding activity of MtrA. **A.** A 526 bp DNA fragment corresponding to the *oriC* was PCR amplified from *M. tuberculosis* H37Rv genomic DNA template using specific primers. EMSA was performed using MtrA treated with various agents, as indicated. The concentration gradient indicates the amount of MtrA used in the assay. **B.** EMSA with *rpfB* gene promoter region using MtrA. The concentration gradient indicates the amount of MtrA used in the assay. **C-G. Effect of acetylation DNA binding ability of MtrA on *oriC* promoter**. Acetylation was performed using acetyl phosphate (AcP) as the donor. Acetylated and non-acetylated wild type MtrA proteins are used as controls. **C** Analysis of DNA binding activities of all three lysine defective mutants (K108R, K110R, and K207R) in the presence and absence of AcP; **D.** Analysis of K108Q lysine mimic mutant; **E.** K110Q or K207Q lysine mimic mutants in the absence of AcP. Analysis of **F.** MtrA K110RK207R (defective) and **G.** K110QK207Q (mimic) double mutants in the presence or absence of AcP. All the experiments were performed as described in the materials and methods section**.** N = 3.

Supplementary Figure 4.

A.

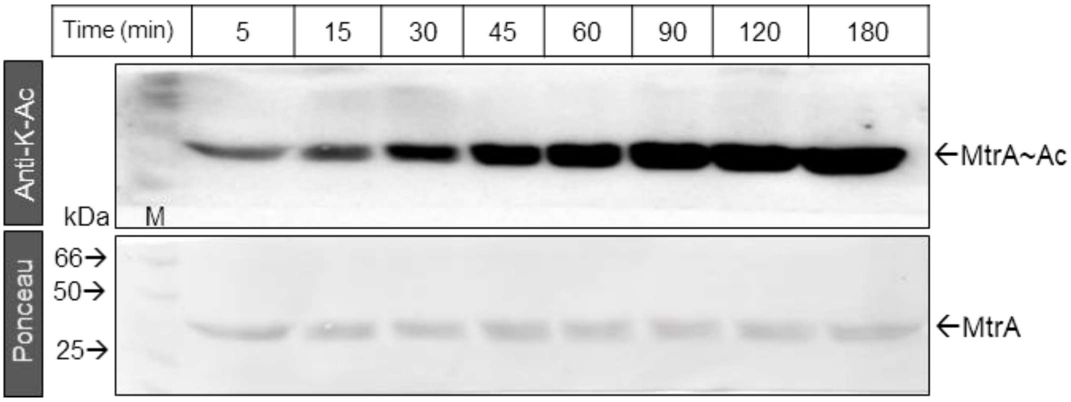

B.

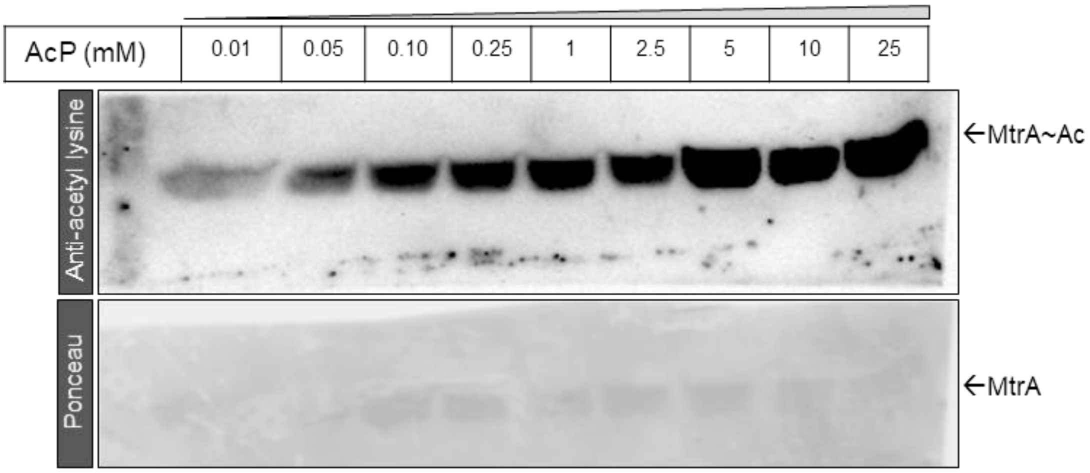

Supplementary Figure 4. A. MtrA acetylation analysis using 5 mM AcP over a time course. This was performed in parallel with EMSA described in Figure 3A by anti-acetyl western blot analysis. B**.** MtrA acetylation analysis with various concentrations of AcP. The analysis was done in parallel with the EMSA experiment depicted in Figure 3E. All the experiments were performed as described in the materials and methods section**.** N = 3.

Supplementary Figure 5.

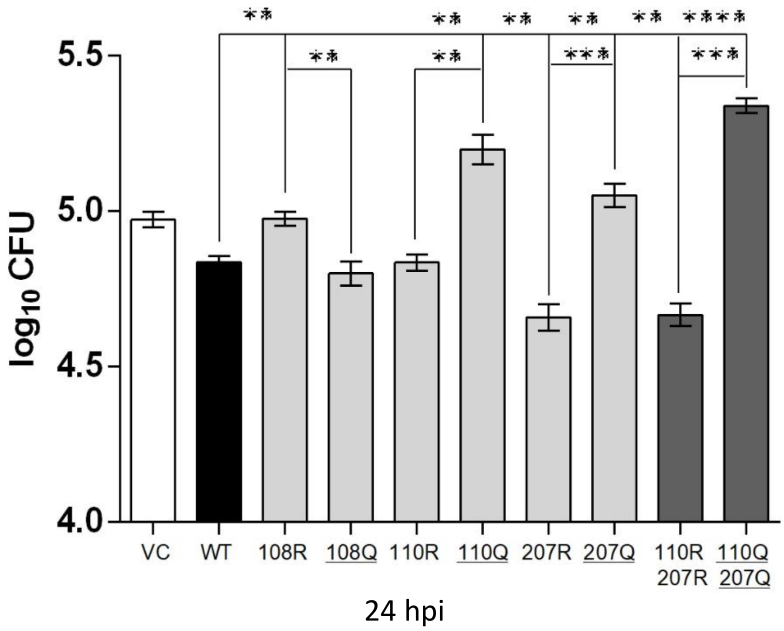

Supplementary Figure 5. Infection was performed in A549 alveolar epithelial cells with various H37Ra strains containing pMV261 plasmid with various variants of MtrA (as indicated) (VC; vector control), following which CFU was determined at 24 hours post-infection. Log_{10} CFU was further calculated for each strain and compared with each other. *P*- values were <0.05 for R and Q mutants by Student's *t*-test.

1. Supplementary methods:

1.1. Protein expression and purification. For protein overexpression, *E. coli* Arctic strain containing the expression plasmids for the sensor kinase and response regulator proteins (Agrawal et al., 2015), GFP-tagged MtrB (Table 2), KATms (MSMEG_5458) (Nambi et al., 2010) were grown at 37°C in 200 ml of 2×YT (Tryptone, 20 gm/L; Yeast extract, 10 gm/L; NaCl, 10 gm/L) or Terrific broth (TB) (Tryptone, 12 gm/L; Yeast extract, 24 gm/L; KH_2PO_4, 2.3 gm/L; K_2HPO_4, 12.5 gm/L; Glycerol, 0.4%) (Singh et al., 2019a) to an OD_{600}>1.0 followed by induction with IPTG (0.1-1.0 mM). The culture was further grown for 15-20 h with shaking at 10-13°C for protein expression. Cells were harvested by centrifugation and stored at -80°C until use. For purification of 6×His tagged proteins from soluble conditions, the protocol described previously was followed (Nambi et al., 2010; Agrawal et al., 2015), described in detail in the section below.

1.2. Purification of soluble proteins using Ni^{+2}-NTA affinity chromatography. Purification of 6xHis-tagged recombinant protein has been reported earlier by Singh et al. (Singh et al., 2014, 2019a). In brief, the *E.coli* cell pellet was resuspended in native lysis buffer (50 mM Tris.Cl, pH 8, 300 mM NaCl, 10 % glycerol) containing 1 mM PMSF and 1 mM benzamidine, sonicated on ice for 5-10 minutes at 25 % amplitude (pulse on 3s, pulse off 2s). The lysate was centrifuged at 12000 rpm for 30 mins at 4°C. The supernatant containing the protein was loaded into the Ni^{2+}-NTA column pre-equilibrated with a native lysis buffer at 4°C for around 15 min. After 120 minutes of incubation with intermittent shaking, the unbound proteins were collected as flow through followed by washing the column with wash buffer A (25 mM Tris.Cl pH 8.0, 500 mM NaCl, 25 mM imidazole, 10% glycerol) and wash buffer B (25 mM Tris.Cl pH 8.0, 500 mM NaCl, 50 mM imidazole, 10% glycerol). The protein was then eluted using the elution buffer (25 mM Tris.Cl pH 8.0, 500 mM NaCl, 250 mM imidazole, 10% glycerol).

1.3. Dialysis and storage of purified proteins. The elution fractions of proteins with the highest yield determined by Bradford's assay were pooled together and dialyzed against dialysis buffer I (50 mM Tris.Cl, pH 8.0, 50 mM NaCl, 1 mM DTT, 10% glycerol) overnight and then with dialysis buffer II or storage buffer (50 mM Tris.Cl, pH 8.0, 50 mM NaCl, 0.1 mM DTT, 50% glycerol) for 6-12 hrs. After dialysis, the protein concentration was determined by Bradford assay using BSA as standard, and purity of the proteins was checked on SDS-PAGE. The purified proteins were stored at -20°C.

1.4. Phosphorylation assays. Purified SKs (50 pmoles of MtrB and MtrB-GFP proteins) was autophosphorylated in the reaction buffer containing 50 mM Tris-Cl (pH 8.0), 50 mM KCl, 20 mM $MgCl_2$, 100µM ATP and 0.2 µCi of [γ^{32}P] ATP (>4000 Ci/mmol, BRIT, India) for 60 min at 30°C. In the phosphotransfer assay, 150 pmoles of the RRs diluted in kinase buffer were added to the reaction containing autophosphorylated SK, followed by incubation at 30°C for 5 min or as otherwise indicated. The reaction was terminated by mixing 1x SDS-PAGE gel loading buffer, and the sample was resolved on an SDS-PAGE gel. After electrophoresis, the gels are washed with dH_2O and exposed to a phosphorscreen (Fujifilm, Japan), followed by imaging with Typhoon phosphorimager (GE Healthcare, USA). Post-acquisition processing of images for brightness and contrast was performed using the Microsoft image editing tool. Quantitative densitometric analysis of the autoradiograms or western blots was done using ImageJ software. For quantitating effects of acetylation, the phosphotransfer signal on the control RR was considered 100%, and relative levels of phosphorylation on the acetylated RR either from cognate SKs were determined. Statistical analysis and significance (p values) were calculated with reference to RR~P levels in the phosphotransfer reaction where maximum levels are recorded.

1.5. Acetylation status analysis. For *in vitro* acetylation, test proteins (1 µg or otherwise mentioned) were incubated in a reaction buffer containing 25 mM Tris-Cl (pH 7.4), 100 mM NaCl, and 5 mM acetyl phosphate at 37°C for 4 hours. The reactions were terminated by boiling and analyzed by western blotting using anti-acetyl lysine antibody (1:7500, #9441, Cell Signaling Technology Inc,

USA), followed by probing with HRPO-conjugated secondary antibody (1:5000) and developed with ECL+ reagent (Perkin Elmer, USA) as per manufacturer's protocol.

1.6. Acetylation site identification by MS/MS analysis. Acetylated proteins were resolved by SDS PAGE electrophoresis, stained with CBB, and bands were incised and trypsinized in gel using standard methods. The gel pieces were destained using a wash solution (50% acetonitrile, 100 mM ammonium bicarbonate), followed by dehydration with 100 % acetonitrile and drying. The gel pieces were treated with a reduction solution (10 mM DTT, 100 mM ammonium bicarbonate) for 30 minutes at 56°C, followed by alkylation (10 mM iodoacetamide, 100 mM ammonium bicarbonate) in the dark for 30 minutes. Gel pieces were then washed, dried, and rehydrated in 50 mM ammonium bicarbonate containing 200-400 ng trypsin (Promega, USA) and incubated overnight at 37°C. Peptide extraction was performed using 60 % acetonitrile, 0.1 % TFA in an agitated water bath, followed by MS/MS analysis.

For MS/MS, peptide extracts were passed through reverse phase C18 column (HPLC1100 Agilent, Zorbax, 4.6×150 mm) coupled to ESI ion trap (HCT UltraII, Bruker Daltonics, Germany) for 55 minutes using a linear gradient of 5-95 % of 0.1% formic acid in acetonitrile and water having the flow rate 0.2 ml/ minute. The dry gas flow rate was used at 10 l/minute and a nebulizer pressure of 30 psi at 300°C. Data was acquired using esquire control and, in the m/z range from 100 to 2600 m/z. For MS-MS, the 3 most intense peaks were auto-selected. The analysis was done using the Mascot server taking carbamidomethyl (C) as a fixed modification, oxidation (M) and acetyl (K) as variable modification. Peptide tolerance was kept as 1.2 Da, and MS/ MS tolerance was maintained as 0.6 Da. To confirm the results, a manual analysis was performed using Data analysis version 4, which established the MS peaks and MS/MS fragmentation representing b and y ions.

1.7. Determination of affinities of RR with AcP using microscale thermophoresis (MST) analysis. Purified recombinant MtrA-RFP protein (50 nM) was incubated with indicated concentrations acetyl phosphate (0.5 nM to 10 µM). The sample was then added into pretreated capillaries and examined using a Monolith NT.115 system (NanoTemper Technologies, GmbH). The blue laser was used for 35 s for excitation (MST power = 60%, LED power 40%). The data were analysed using MO Control software (NanoTemper Technologies, GmbH) to ascertain the dissociation constants (KD) for interacting proteins.

1.8. Electrophoretic mobility shift assay (EMSA). For EMSA, the labelled DNA was incubated with the indicated amounts of MtrA protein for 30 min (or as otherwise indicated) at 25°C in the binding buffer (25 mM Tris.Cl pH 8.0, 20 mM KCl, 6 mM $MgCl_2$, 0.10 mg/ml BSA, 0.5% glycerol, 1 mM DTT, 0.5 mM EDTA and 1µg poly dI.dC). MtrA~P was obtained by incubating autophosphorylated MtrB~P in a buffer containing 50 mM Tris.Cl (pH 8.0), 50 mM KCl, 20 mM $MgCl_2$, 5 mM ATP. Subsequently, the reaction mixtures were resolved on a 4% native polyacrylamide gel (29.5:0.5), pre-equilibrated for 1-2 h at 80 V in 0.5× Tris-borate EDTA buffer at 4°C. Electrophoresis was performed at 4°C at 80 V for 2-3 h. The DNA-protein complexes were visualized by phosphorimaging, as described above.

1.9. Fluorescence resonance energy transfer (FRET). Wells of 96-well flat, black transparent bottom plate (Corning) were pre-treated with 200 µl of 1:1 methanol: HCl for 10 min, after which it was washed thrice with the same volume of MQ water (Agrawal et al., 2016). These wells were later incubated with 50 µl of 10 mg/ml BSA solution for 15-30 min. Wells were further washed gently with 200 µl water and then incubated with 200 µl of kinase buffer.

Proteins to be used for FRET analysis were diluted in 1x kinase buffer and added to the pre-treated wells for fluorescence measurements. Tagged SK MtrB-GFP (2.5µM) and RR MtrA-RFP (10 µM) were used for FRET. Fluorescence intensities were recorded in the following channels, DD (donor excitation donor emission) channel (505 nm excitation and 515 nm emission); DA (donor excitation acceptor emission) channel (505 nm excitation and 600 nm emission), using the bottom reading mode in the Infinite M1000 PRO multimode fluorescence plate reader (Tecan, Austria). Fluorescence

intensities were measured before and after the addition of MtrA-RFP acceptor in the presence or absence of 5 mM ATP/ 5 mM acetyl phosphate. Graphs were plotted as a ratio of fluorescence intensities obtained in the DA channel by that of the DD channel (DA/DD) as a function of time. The blank sample's fluorescence well and the buffer only wells were subtracted from all corresponding test wells.

1.10. *In vivo* acetylation analysis of MtrA in *E.coli.* To determine if MtrA is acetylated by acetyl phosphate *in vivo*, the phosphotransacetylase (Δ*pta*) and acetate kinase (Δ*ackA*) knockout BL21 *E. coli* strains were transformed with pProExHtb: MtrA expression vector. The single colony was inoculated for the primary culture, and subsequently, the secondary culture was grown to 0.4-0.6 OD_{600}. The culture was induced with 0.5 mM IPTG and incubated for 11-13°C for 3-4 hours. Furthermore glucose (final concentration 1 %) or sodium acetate (final concentration 2.5 %) was added and incubated for 3-4 hour at 11-13°C. Subsequently, cells were then harvested by pelleting down and resuspended the cells in 500 µl of lysis buffer (50 mM Tris-Cl (pH ~ 8.0), 300 mM NaCl, 1 mM PMSF, 1 mM Benzamidine hydrochloride). Cells were then sonicated for 30 sec at 10% amplitude at 2sec ON, 3sec OFF. The sonicated sample is centrifuged at 12000 rpm for 5 min at 4°C collected; the supernatant and protein was estimated by Bradford's reagent. Acetylation status and Anti-6xhis were analyzed by western blotting as per the protocol described above or described previously (Singh et al., 2019b, 2019a).

1.11. *In vitro* growth analysis. To determine the effects of overexpression of MtrA or various mutants on *M. tuberculosis* growth in nutrient-rich media and nutrient starvation conditions. Strains carrying plasmids with MtrA protein with mutations generated by SDM in vector pMV261: *mtrA* were used. Vector alone or vector containing wild type or mutant *mtrA* genes (as indicated) were electroporated into *M. tuberculosis* H37Ra and were plated on 25µg/ml kanamycin containing Middlebrook 7H11-OADC agar plate. Bacteria were grown into the mid-log phase in Middlebrook 7H9-OADC-Tween 80 media for the primary culture. Secondary cultures of each strain were prepared with an equal starting OD_{600}. The strains were grown in rich (7H9-OADC-Tween 80) or poor (7H9-Tween 80) media only. Bacterial growth was monitored by measuring absorbance at 600nm every day.

1.12. Infection analysis *in vivo* in A549 cells. Infection experiments were performed using A549, an alveolar epithelial cell lines, as host. Briefly, the alveolar epithelial cell line, A549, was maintained in Dulbecco's Modified Eagle Medium (DMEM) containing 10% FBS before starting the experiment. Cells were seeded (10^5 cells per well) in 12-well tissue culture plates and allowed to grow to 50% confluency. These were then infected at an MOI of 1:10 with various bacterial strains. The bacteria could infect cells for 4 hours (taken as the time point of invasion), after which cells were either lysed with 0.01% Triton X-100 and plated after serial dilutions on Middlebrook 7H11-OADC (BD) plates for colony enumeration. For intracellular growth analysis, the washed cells were supplemented with fresh DMEM medium without antibiotics and lysed at a later time point of 24 hours and plated for CFU analysis. This was repeated to obtain three biological replicates.

Table 1. List of primers used in the present study.

Primers	Sequence	Reference
mtrBF (MtrB-GFP)	5′CGAGGACGCCATGGCCAGG3′	(Singh et al., 2019a)
mtrBR (MtrB-GFP)	5′GATCCTCGAGACCGCTCCACTCCGCGTGCT3′	(Singh et al., 2019a)
gfpF (MtrB-GFP)	5′GATCCTCGAGGGATCTGGTGGAGGTATGGTGAGCA AGGGCGAGGAG3′	(Singh et al., 2019a)
gfpR (MtrB-GFP)	5′GATCCTCGAGACCGTCCACTCCGCGTGCT3′	(Singh et al., 2019a)
mtrAF (MtrA-RFP)	5′GACACCATGGACACCATGAGG3′	This study
mtrAR (MtrA-RFP)	5' GATCCTCGAGCGGAGGTCCGGCCTTGTACC 3'	This study
*rfp*F (MtrA-RFP)	5′GATCCTCGAGGGGATCTGGTGGAGGTATGGTGTCT AAGGGCGAAGAGC3′	This study
*rfp*R (MtrA-RFP)	5′GATCAAGCTTTCAATTAAGTTTGTGCCCCAGTTTG3′	This study
rpfB proF	5′GATCGAATTCTCCGTACCGCCCGTGAAC3′	(Sharma et al., 2015)
rpfB proR	5′GATCGTCGACCATCGGTGATTGGGTC 3′	(Sharma et al., 2015)
oriC proF	5′ CATCCGTCAGCGCTCC 3′	(Purushotham et al., 2015)
oriC proR	5′ TTGCGCCCTTTCACCTCACGATG 3′	(Purushotham et al., 2015)
mtrA K108RF	5′ GAAGCCGTTCAGGCCCAAGGAGCTGG 3′	This study
mtrA K108RR	5′ CCAGCTCCTTGGGCCTGAACGGCTTC 3′	This study
mtrA K108QF	5′ GAAGCCGTTCCAGCCCAAGGAGCTGG 3′	This study
mtrA K108QR	5′ CCAGCTCCTTGGGCTGGAACGGCTTC 3′	This study
mtrA K110RF	5′ CGTTCAAGCCCAGGGAGCTGGTTGCG 3′	(Singh et al., 2019a)
mtrA K110RR	5′ CGCAACCAGCTCCCTGGGCTTGAACG 3′	(Singh et al., 2019a)
mtrA K110QF	5′ CGTTCAAGCCCCAGGAGCTGGTTGCG 3′	This study
mtrAK110QR	5′ CGCAACCAGCTCCTGGGGCTTGAACG 3′	This study
mtrAK207RF	5′ GGCCAAGGTCGAAAGGGATCCCGAGAACC 3′	This study
mtrAK207RR	5′ GGTTCTCGGGATCCCTTTCGACCTTGGCC 3′	This study
mtrA K207QF	5′ GGCCAAGGTCGAACAGGATCCCGAGAACC 3′	This study
mtrA K207QR	5′ GGTTCTCGGGATCCTGTTCGACCTTGGCC 3′	This study

Table 2. List of Plasmids and knockout strains used in this study

**Plasmids**	**Source**	**Reference**
Rv3246c, MtrA cloned in pPROEx-HT vector	Lab collection	Agrawal et al., 2015
Rv3245C, MtrB cloned in pPROEx-HT vector	Lab collection	Agrawal et al., 2015
Rv1027c, KdpE cloned in pPROEx-HT vector	Lab collection	Agrawal et al., 2015
MSMEG_5458, KATms	Gift	(Nambi et al., 2010
MtrBGFP-pProEx-HTc	This study	
MtrA-RFP-pProEx-HTb	This study	
MtrAK108R- pProEx-HTb	This study	
MtrAK108Q- pProEx-HTb	This study	
MtrAK110R- pProEx-HTb	Lab collection	Singh et al., 2019a
MtrAK110Q- pProEx-HTb	This study	
MtrAK207R pProEx-HTb	This study	
MtrAK207Q- pProEx-HTb	This study	
MtrAK110R K207R- pProEx-HTb	This study	
MtrAK110Q K207Q- pProEx-HTb	This study	
pMV261	Lab collection	Singh et al., 2019a
pMV261-MtrA	This study	
pMV261-MtrAK108R	This study	
pMV261-MtrAK108Q	This study	
pMV261-MtrAK110R	This study	
pMV261-MtrAK110Q	This study	
pMV261-MtrAK207R	This study	
pMV261-MtrAK207Q	This study	
E.coli MG1655 mutant Phosphotransacetylase-Δ*pta*	Gift	Weinert et al., 2013
E.coli MG1655 mutant acetate kinase –Δ*ackA*	Gift	Weinert et al., 2013
E.coli BL21 arctic mutant phosphotransacetylase Δ*pta*	This study	
E.coli BL21 mutant acetate kinase –Δ*ackA*	This study	

3. References

Agrawal, R., Kumar V, P., Ramanan, H., and Saini, D. K. (2016). FRET reveals multiple interaction states between two component signalling system proteins of M. tuberculosis. *Biochim. Biophys. Acta* 1860, 1498–507. doi:10.1016/j.bbagen.2016.04.011.

Agrawal, R., Pandey, A., Rajankar, M. P., Dixit, N. M., and Saini, D. K. (2015). The two-component signalling networks of *Mycobacterium tuberculosis* display extensive cross-talk *in vitro*. *Biochem. J.* 469, 121–134. doi:10.1042/BJ20150268.

Nambi, S., Basu, N., and Visweswariah, S. S. (2010). cAMP regulated Protein Lysine Acetylases in Mycobacteria. *J. Biol. Chem.* 285, 24313–24323.doi:10.1074/jbc.M110.118398.

Purushotham, G., Sarva, K. B., Blaszczyk, E., Rajagopalan, M., and Madiraju, M. V. (2015). *Mycobacterium tuberculosis oriC* sequestration by MtrA response regulator. *Mol. Microbiol.* 98, 586–604. doi:10.1111/mmi.13144.

Sharma, A. K., Chatterjee, A., Gupta, S., Banerjee, R., Mandal, S., Mukhopadhyay, J., et al. (2015). MtrA, an essential response regulator of the MtrAB two-component system, regulates the transcription of resuscitation-promoting factor B of Mycobacterium tuberculosis. *Microbiology* 161, 1271–81. doi:10.1099/mic.0.000087.

Singh, K. K., Bhardwaj, N., Sankhe, G. D., Udaykumar, N., Singh, R., Malhotra, V., et al. (2019a). Acetylation of Response Regulator Proteins, TcrX and MtrA in M. tuberculosis Tunes their Phosphotransfer Ability and Modulates Two-Component Signaling Crosstalk. *J. Mol. Biol.* 431, 777–793. doi:10.1016/j.jmb.2019.01.004.

Singh, K. K., Jain, R., Ramanan, H., and Saini, D. K. (2014). Matrix-Assisted Refolding, Purification and Activity Assessment Using a -Form Invariant Assay for Matrix Metalloproteinase 2 (MMP2). *Mol. Biotechnol.* 56, 1121–1132. doi:10.1007/s12033-222 014-9792-7.

Singh, K., Singh, D., Singh, R., and Saini, D. (2019b). In vitro and in vivo Assessment of Protein Acetylation Status in Mycobacteria. *BIO-PROTOCOL* 9. doi:10.21769/BioProtoc.3291.

Weinert, B., Iesmantavicius, V., Wagner, S., Schölz, C., Gummesson, B., Beli, P., et al. (2013). Acetyl-Phosphate is a critical determinant of Lysine Acetylation in E.coli. *Mol. Cell* 51, 265–272. doi:10.1016/j.molcel.2013.06.003.
